# Latitudinal and elevational variation in the reproductive biology of house wrens, *Troglodytes aedon*

**DOI:** 10.1002/ece3.10476

**Published:** 2023-09-12

**Authors:** Rachel N. Levin, Stephanie M. Correa, Kate A. Freund, Matthew J. Fuxjager

**Affiliations:** ^1^ Department of Biology Pomona College Claremont California USA; ^2^ Department of Neuroscience Pomona College Claremont California USA; ^3^ Present address: Department of Integrative Biology & Physiology University of California Los Angeles CA 94720 USA; ^4^ Present address: US Fish & Wildlife, Pacific Region Portland Oregon USA; ^5^ Present address: Department of Ecology, Evolution, and Organismal Biology Brown University Providence Providence RI 02906 USA

**Keywords:** avian reproduction, breeding, elevation, house wren, latitude, life history, parental behavior, Troglodytes aedon

## Abstract

While cross‐species comparisons of birds suggest that as latitude decreases or elevation increases, clutch size decreases and the duration of developmental stages and parental attentiveness increases, studies comparing populations of the same species are rare. We studied populations of house wrens, *Troglodytes aedon*, at high and low elevations in California and Costa Rica, collecting data on clutch size, the duration of incubation and nestling periods, parental attentiveness, nestling growth rate, and nesting success. Our data support results from cross‐species comparisons, but also revealed unanticipated results from low elevation temperate zone house wrens in the southwest. This population had prolonged incubation and nestling periods similar to those found in the tropics. We also found that temperate zone females, especially those at our higher elevation site, spent more of their day incubating than did tropical females. Nest temperature at our high elevation temperate zone site was higher than that at all other tropical sites. Age at fledging did not differ between sites. Total feeding rates per chick and male feedings per chick did not vary between sites. Nest success rates showed the predicted effect of latitude, but not the predicted effects of elevation. Our results extend low elevation house wren research into the southwestern US and contribute the first intraspecific elevational comparison in the Neotropics. Data from our low elevation southwestern site present a unique suite of life history traits that align more with tropical house wrens, although with a larger clutch size, and point to food limitation and/or high predation pressure as being possible drivers of some of these differences. These results highlight the need for additional studies of house wrens and other broadly distributed species at a more diverse array of sites to better understand which forces drive the evolution of different life history strategies across major biogeographical gradients.

## Graphical abstract

This study presents the first comparison of the reproductive biology of a single species, the house wren (*Troglodytes aedon*), at high and low elevations at temperate and tropical latitudes. It extends extant research on this species into the southwestern US and provides the first comprehensive data with which to compare reproductive biology at different elevations in the Neotropics. Results from the low‐elevation southwest population differ from results of studies conducted elsewhere in the US and align more with tropical wrens. These results call for more diversity in the selection of study populations and provide new insight into the selective pressures underlying life history evolution.

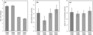

## 1 INTRODUCTION

With limited resources, organisms are forced to balance their time and energy between processes related to both reproduction and survival. Life history theory provides a framework to explore this balance by generating a set of rules that suggest how organisms have evolved to optimally allocate their resources in different environments (Roff, 1992; Stearns, 1992). Yet, developing these rules can be challenging, particularly if the goal is to derive principles that apply broadly across large geographic areas and taxonomic groups.

Research on birds has played a significant role in the development of life history theory (Lack, 1948, 1954, 1968; Martin, 2004; Moreau, 1944; Ricklefs, 2000; Roff, 1992). Observations that tropical birds have smaller clutches than temperate birds, longer duration of incubation and nestling periods, increased parental attentiveness, and higher predation rates (Chalfoun & Martin, 2007; Gill & Hagerty, 2012; Jetz et al., 2008; Lack, 1948, 1954, 1968; Martin, 2004; Moreau, 1944; Robinson et al., 2010) led to the hypotheses that factors such as nest predation, food limitation, and adult and/or chick survival drive the evolution of different life history strategies (for a review, see Gill & Hagerty, 2012; Llambias et al., 2015; Martin, 1995). More recent work added consideration of variation in physiological and metabolic traits (Ricklefs & Wikelski, 2002), resulting in the “pace‐of‐life hypothesis,” which is based on the insight that species can be arranged on a continuum of slow‐paced (e.g., many tropical species) to fast‐paced species (e.g., many north temperate species; Dammhahn et al., 2018; Wikelski et al., 2003). Most studies compare species, often of closely related groups, in north temperate latitudes to those in tropical latitudes (e.g., Arslin & Martin, 2019; Austin et al., 2020; Boyce et al., 2020; Boyce & Martin, 2017; Geffen & Yom‐Tov, 2000; Martin, 1995, 2004), with many noting the relative paucity of complete datasets from tropical species. Expansion of this work to include species in the south temperate zone has reported similarity in life historytraits between south temperate and tropical birds (Auer et al., 2007; Llambias et al., 2015; Martin, 1996; Moreau, 1944; Peach et al., 2001, Russell et al., 2004; Yom‐Tov et al., 1994).

Effects of elevation on life history traits have received far less attention than effects of latitude, and, further, variation in elevation effects between different latitudes has not been directly examined. Nonetheless, several cross‐species comparisons (Badyaev, 1997; Badyaev & Ghalambor, 2001; Boyce et al., 2015) and studies of single species at different elevations (Bears et al., 2008; Camfield et al., 2010; Johnson et al., 2018; LaBarbera & Lacey, 2018; Lundblad & Conway, 2019; Tieleman, 2009; Wilson & Martin, 2011) suggest that an increase in elevation is correlated with larger birds, smaller clutches, longer incubation periods, and increased male participation in provisioning (but see Hille & Cooper, 2015). Thus, because high‐elevation birds seem to show the same traits as tropical birds, they have been characterized as having the same slower pace of life seen in tropical birds, and it was suggested that the same selective pressures, with the addition of colder temperatures and shorter breeding seasons, were driving this strategy. However, a meta‐analysis by Boyle et al. (2015) compared available data on low‐ and high‐elevation populations from studies involving 101 species of birds and concluded that while clutch size decreased and duration of chick development increased with an increase in elevation, not all species exhibited all of the characteristics of the slow‐pace strategy (e.g., Evans‐Ogden et al., 2012). The results of this study led the authors to reject predation and colder temperature as driving factors at high elevations and to suggest that either food availability (or its temporal constraints) or temperature (interacting with another factor) might drive the array of traits seen in high‐elevation birds. In so doing, the authors noted that most studies did not record a wide variety of traits as well as noting a limit in the geographical location of species that have been studied. In a meta‐analysis that focused specifically on elevational effects on clutch size in tropical birds, Boyce et al. (2015) also reported both that clutch size decreased with elevational gradients and reported geographic variation in results. The authors of these two meta‐analyses called for more studies in the tropics, and more specifically, tropical studies focusing on elevational effects.

While the results of comparisons of related species at different latitudes and elevations may show a dominant pattern, meta‐analyses, and general reviews (e.g., Hille & Cooper, 2015) point to variations from expected patterns, a problem that can be addressed by comparing populations of the same species across different locations (Boyce et al., 2015). Unfortunately, intraspecific comparisons of populations at different latitudes or elevations are relatively uncommon. The house wren, *Troglodytes aedon*, offers a relatively unique opportunity for comparative work as it is distributed throughout North, Central, and South America at a variety of elevations,although its status as a single species has been called into question (Klicka et al., 2023). As such, it has received a fair amount of attention by those seeking to understand how best to explain life history strategies. House wrens have been the subject of several long‐term studies in the United States (Baltz & Thompson, 1988; Johnson, 2020; Kendeigh, 1941; Robinson & Rotenberry, 1991), in part because they conveniently prefer to use artificial nest boxes that researchers place in the field (Johnson, 2020). Nest boxes can increase clutch size and hatching and fledging success and decrease predation rates (Purcell et al., 1997). Nonetheless, with careful consideration of box placement to avoid increasing natural population density (e.g., Hagvar et al., 1990), the use of nest boxes facilitates easy observation and comparison between sites.

Comparisons between north temperate and tropical house wren reproductive biology have focused on clutch size (Robinson et al., 2008; Young, 1994), but confirm that house wrens in the tropics have larger body size, smaller clutches, longer duration of incubation and nestling periods, and fewer incubation visits than do those in the north temperate zone (Johnson, 2020). More recent studies of South temperate house wrens have included a consideration of physiology (Tieleman et al., 2006) and behavior (Llambias et al., 2015; Tieleman et al., 2006) and suggest that south temperate wrens are more similar to tropical wrens than to those in the north temperate zone (Johnson, 2020).

Little attention has been given to elevation as a factor in house wren life history traits and, as is the case with studies of other species, no attention has been given to how elevation effects might differ between temperate and tropical populations. Young (1994) studied tropical house wens at four elevations, but focused on food availability, climate, the onset and length of the breeding season, and predation rates; clutch size was only reported for one site (Young, 1996), and duration of incubation and fledging was not reported. Most studies of north temperate house wrens occur at elevations of 2–500 m in the midwestern and eastern US, with only two low‐elevation studies in the west (Purcell, 2011; Young, 1994). Two studies of house wrens were conducted at higher elevations, all in the west—one in Wyoming (1310 m, Johnson, 2020) and one in Arizona (2600 m, Palacios & Martin, 2006), but only the study in Wyoming reported an array of life history traits. These studies suggest, at the very least, that the duration of the nestling period increases with an increase in elevation. One unpublished study directly compared size and developmental traits at two elevations in Colorado and found the expected slower pace‐of‐life pattern in higher elevation birds (Milinkovich, 1993).

We expanded upon previous studies by studying populations of house wrens in Southern California and Costa Rica, doing so at two elevations for each latitude. Our sites expand the US range of house wren study populations into the far west, add a consideration of elevation in tropical wrens, and provide one of the first studies of latitudinal and elevational intraspecific variation that includes comprehensive data on body and egg size, clutch size, duration of developmental stages, and parental attentiveness.

## 2 MATERIALS AND METHODS

### 2.1 Study populations and box monitoring

We studied house wrens at four sites chosen as part of a larger study of variation in song and hormone levels. In the temperate zone, we studied house wrens for their entire breeding season (first to last clutch initiations) at two locations in California. In both the southern California lowlands and the Eastern Sierras, we found house wrens to be distributed in relatively small populations, associated with lakes and streams, presumably due to drier, warmer conditions. We studied a high‐elevation population from May to early July 1997–2003 and 2005 at the Sierra Nevada Aquatic Research Laboratory (SNARL, “temp‐high”), 13 km east of Mammoth Lakes, CA, from 1997 to 2003, and in 2005 (37.614° N, 118.833° W, elevation: 2164 m, *n* = 73 boxes). Here, house wrens were primarily associated with small willow‐ and aspen‐lined streams. Our low‐elevation site was located 396 km south in Marshall Canyon (“temp‐low”) in La Verne, CA (34.153° N, 117.749° W, elevation = 480 m, *n* = 83 boxes). Here, we conducted observations throughout the entire breeding season, from late March through mid‐July, 2003 to 2008. This site experiences a Mediterranean climate and consists of riparian oak woodlands and coastal sage scrub. Marshall Canyon is a refuge for house wrens and other birds, as well as large and small mammals, as it is one of the few canyons in the region with a year‐round small stream. While our high and low sites differ slightly in latitude, their difference is below the 5° threshold used by Boyle et al. (2015) for between‐elevation comparisons.

Our tropical sites were located in Costa Rica. In the tropics, house wrens are likely to be found in disturbed habitats and have been studied at two lowland sites in Panama—cattle ranches (Freed, 1981), and a national park edge (Robinson et al., 2008), as well as in cattle ranches in and near the cloud forests of Costa Rica (Young, 1994). For our high‐elevation site, we worked on cattle ranches in the cloud forests of San Luis (“trop‐high,” 10.282° N, 84.799° W, elevation = 1342 m, *n* = 72 boxes) in 2001 and 2004. Our low‐elevation site was located 87 km east in cattle ranches at the edge of lowland tropical rainforest near La Selva Biological Station in Puerto Viejo de Sarapiquí (“trop‐low,” 10.422° N, 84.015° W, elevation = 35 m, *n* = 82 boxes). Here, observations were conducted in 2002 and 2004. At both sites, some birds were breeding before we arrived in mid‐January of each year, and some were still breeding when we left in mid‐July; our field seasons covered the breeding peak at both sites.

During the first year at each site, nest boxes were attached to trees at each location where a house wren was singing. Boxes were replaced as needed in the second observation year. The second year at each tropical site (2004) was used for cross‐site comparisons, since boxes were in place before breeding began. The same year was selected for these comparisons for our temp‐low site, except for egg weights as noted below. Data were not collected in 2004 from our temp‐high site, so data from 2003 were used in cross‐site comparisons.

No antipredator devices were attached to the boxes. Nest boxes at all sites were checked for evidence of reproductive activity every other day. As predicted dates of hatching and fledging approached, we began checking boxes daily to determine exact dates of hatching and fledging. Incubation duration was calculated using the day of clutch completion as Day 0 through the day the first chick hatched. The duration of the nestling period was calculated with the day of first hatch as Day 0 through the day in which chicks fledged.

As early as possible in the season, adults on each site were mist‐netted, given a unique combination of three colored plastic bands and one numbered aluminum band, weighed, and wing cord and bill measurements taken. Netting was avoided during egg laying; some adults were weighed during nestling feeding if they had not been caught previously. All eggs in a nest were weighed the day after clutch completion. All nestlings were weighed on Days 5 and 10 post‐hatch when possible, and each nestling was given a numbered aluminum band before fledging.

A nesting attempt was considered successful if at least one nestling fledged. In all cases where clutches completely disappeared, evidence suggested either predation by snakes (nest undisturbed, eggs/chicks gone) or mammals (nest destroyed/structure disrupted), but this could not be directly confirmed.

### 2.2 Incubation monitoring

Onset HOBO Pro Series data loggers were attached to a subset of nest boxes at each site before or during the egg‐laying period to monitor female behavior. Ambient temperature probes from each logger were screwed onto the outside bottom of the nest box, and the internal probe was woven through the nest lining until it was at the nest cup surface within 0.5 cm of the eggs. Internal probes were set to maximize detection of arrival and departure of the female from the nest, and are thus likely to provide a relative, but not absolute measure, of nest temperatures at each site. Loggers were programmed to monitor both ambient and internal nest temperature at 30‐second intervals throughout the duration of incubation. The beginning or end of an incubation bout was recorded when the internal temperature probe changed more than 0.5°C within a 30‐s period (Bollinger & Gonser, 2018; Evans‐Ogden et al., 2012). These changes were clearly seen in the temperature records, and these estimates were confirmed and determined to be 100% accurate by simultaneously videotaping and temperature monitoring at least five nest boxes at the beginning of the breeding season at each of our four sites. Nest box and ambient temperatures, total time in the nest box, number of incubation bouts, and incubation bout duration per day for each nest were averaged across the duration of incubation.

### 2.3 Feeding observations

Nest boxes with nestlings were observed by individual researchers on Days 3, 4, 5, 8, 11, and 14 posthatching, following Johnson and Barclay (1996). Observers rotated between boxes; training was conducted to maintain interobserver reliability of 95%. Observations began within 1 h of sunrise and continued for 1 h, noting arrival time, bird ID, and departure time of each parental visit to the nest box. When identifying color band combinations of feeding birds could not be seen, visits were counted in totals, but coded as “unknown ID.” Feeding rates per observation day were totaled as well as adjusted for brood size at each observation.

We used four measures to compare feeding behavior between sites: total feeds per nest, total feeds per nestling, percent of the feedings that were performed by males, and absolute number of feeding visits by males (the latter two measures using data where birds could be identified). For each feeding measure, we calculated “early” and “late” feeding averages in the nestling period by using the mean of observations on Days 3 and 5 (Week 1) and Days 11 and 14 (Week 2), respectively. This analysis only included pairs for which repeated observations were available in the first and second week postfledging.

Each author collected data at both temperate and tropical sites; three of the authors collected data and/or trained and supervised data collection and behavioral observations at all four sites.

Protocols were approved by the Institutional Animal Care Committees of Pomona College and the University of California Santa Barbara. Research in Costa Rica was conducted under permits from the Costa Rican Ministry of the Environment and Energy (MINAE) and overseen by the Organization for Tropical Studies.

### 2.4 Statistical analyses

All analyses were conducted using SPSS 28.0.1.0. Interyear variability in life history parameters for each site was conducted using one‐way analyses of variance and *t*‐tests. A coefficient of variation ([mean/SD] × 100) was also calculated to estimate interyear variability in each parameter at each site. For comparisons between sites, we used the year in which both tropical sites were observed, 2004, and the closest year for which comparable data were available for the temperate sites (2003 for temp‐high; 2004 for temp‐low for all data except egg weights which were first taken at this site in 2005). The one exception was a comparison of nesting success rates where we used all nest data from all years to permit statistical analysis. In this analysis, nests were considered successful if at least one nestling fledged from a completed clutch. This measure of “apparent success” was possible because we were able to monitor all the nests of singing males at each site; this measure is used in comparisons across house wren studies (Johnson, 2020).

To avoid any possible effect of seasonal progression on breeding behavior, only first clutches for which data were available were used in analyses involving clutch size, incubation duration, feeding rates, fledging age, and weights of eggs and chicks. We used mean weights per clutch when comparing chick and egg weight between sites. All data were tested for skewedness and kurtosis before analysis and found to be normally distributed. For behavioral, reproductive, or morphological parameters, we conducted comparisons between sites using a two‐way analysis of variance, with latitude and elevation as fixed factors. A repeated measures two‐way ANOVA was used to analyze feeding behavior across time. If a two‐way ANOVA was significant (*p* ≤ .05), simple slope analyses using pooled variances were used for follow‐up pairwise comparisons.

## 3 RESULTS

### 3.1 Length of breeding season

**FIGURE 1:**
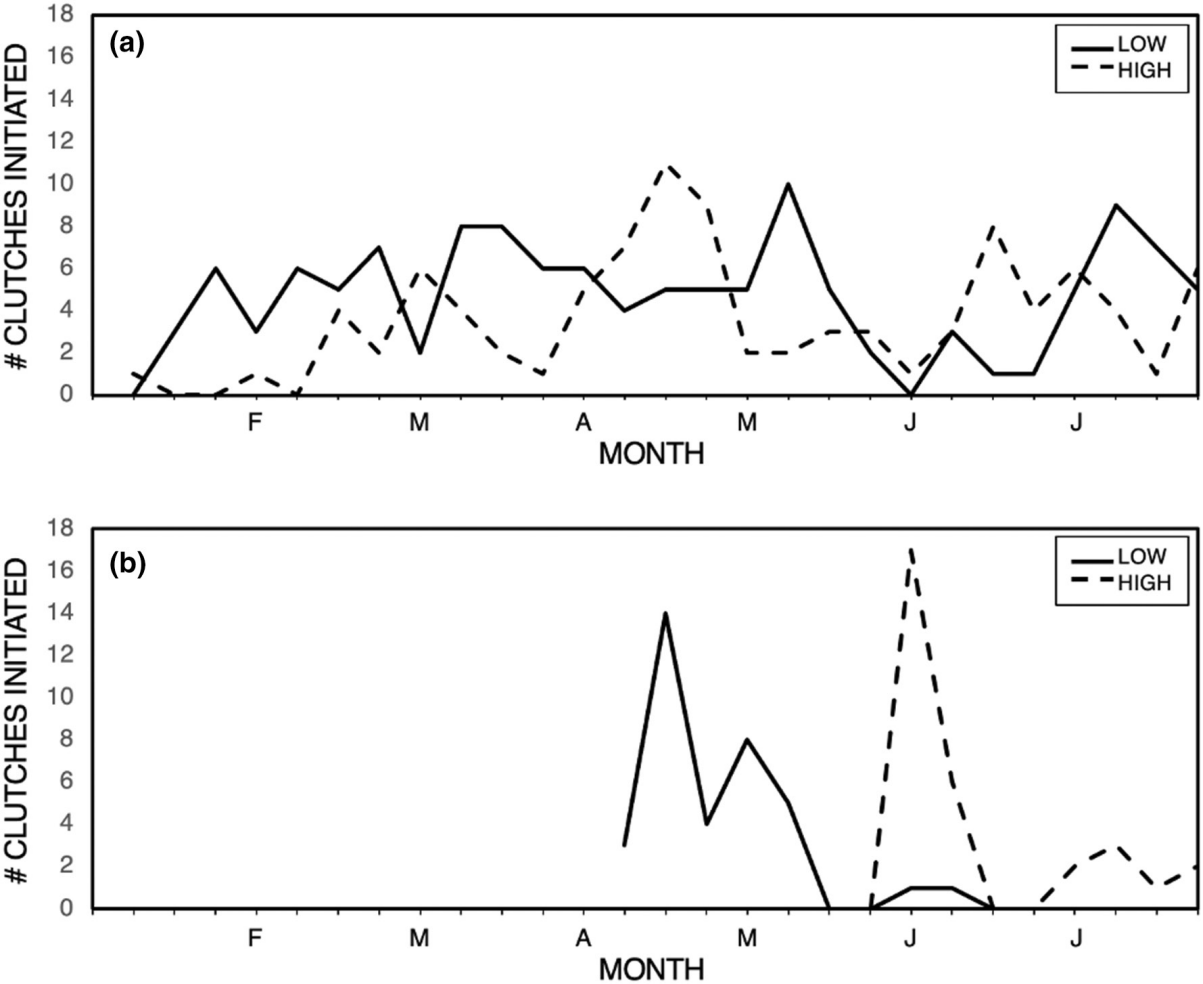
Timing of clutch initiations at tropical (a) and temperate (b) high‐ and low‐elevation sites. See text. Some birds at both high‐ and low‐elevation tropical sites may have initiated clutches before we arrived and some pairs were still breeding when we left.

Length of the breeding season was defined as the number of days between the initiation of the first and last clutch at each site. The breeding season was shorter in the temperate sites than the tropical sites. In addition, in the temperate zone, low‐elevation birds began breeding before high‐elevation birds. Figure 1 displays clutch initiation dates at each site for 1 year (see below), from which the length of the breeding season can be calculated (temp‐low 60 days, temp‐high 56 days, trop‐low >196 days, and trop‐high >181 days). Breeding season onset and duration at our tropical sites is a minimum estimate because one pair was breeding at each tropical site when we arrived in mid‐January (and clutch initiation date could be determined), and a few new clutches were initiated shortly before we left in mid‐July (Figure 1a). Breeding season was observed for its entirety at both temperate sites (Figure 1b). Clutch initiation across 4 years at the low‐temperate site occurred from April 1 (SE = 4.0 days) to June 9 (SE = 6.5 days); across 7 years at the high‐elevation temperate site, clutch initiation occurred from May 20 (SE = 2.0 days) to July 11 (SE = 3.8 days). At all sites, there were two peaks in clutch initiations—a large first peak and a second, much smaller peak; this effect was more pronounced at temperate zone sites (Figure 1).

### 3.2 Breeding parameters

We first analyzed within‐site variation in breeding parameters across years. Clutch size did not differ significantly across years at any one site (temp‐high: *F* = 0.894, *df* = 7, 229, *p* = .512, range of annual means 5.9–6.8; temp‐low: *F* = .344, *df* = 3, 128, *p* = .794, range of annual means 6.6–6.8; trop‐high: *t* = 1.45, *df* = 148, *p* = .149, range of annual means 3.3–3.5; trop‐low: *t* = −1.738, *df* = 154, *p* = .084, range of annual means 3.3–3.5), although the interyear variability in clutch size for our temp‐low site was lower than that for other sites (Table 1). Incubation and nestling periods did not vary between years at the two tropical sites (Table 1; incubation—trop‐high: *t* = −1.868, *df* = 91, *p* = .065, range of annual means 14.7–15.1; trop‐low: *t* = −1.095, *df* = 94, *p* = .276, range of annual means 14.0–14.1; nestling—trop‐high: *t* = −.579, *df* = 56, *p* = .565, range of annual means 18.1–18.3; trop‐low: *t* = .288, *df* = 50, *p* = .664, range of annual means 17.7–17.9), whereas both incubation and nestling periods differed between years at both temperate zone sites (Table 1; incubation, temp‐high: mean range = 12.5–13.4, *F* = 6.019, *df* = 7, 174, *p* < .001, range of annual means 12.5–14.0; temp‐low: mean range = 13.0–14.5, *F* = 12.466, *df* = 3, 131, *p* < .001; range of annual means 13.0–14.3; fledging, temp‐high: mean range = 16.8–17.9, *F* = 2.289, *df* = 6, 134, *p* = .039, range of annual means 16.8–18.2; temp‐low: mean range = 18–19.4, *F* = 6.690, *df* = 3, 91, *p* < .001, range of annual means 18.0–19.4). Coefficients of variation for duration of incubation and the time to fledge indicated higher variability in temperate than tropical bids (Table 1).

**TABLE 1:** Interyear coefficients of variation in breeding parameters.

	Clutch size (eggs)^a^	Incubation (days)^b^	Fledge (days)^b^	Success (% nests)
Temp‐low (4 years)	2.23	4.58	3.41	12.69
Temp‐high (6 years)	4.54	4.34	2.53	18.56
Trop‐low (2 years)	4.16	0.50	0.79	12.63
Trop‐high (2 years)	4.16	1.90	0.77	25.83

^a^ No significant interyear variation for any site.

^b^ Significant interyear variation for temperate, but not tropical sites. See text.

**FIGURE 2:**
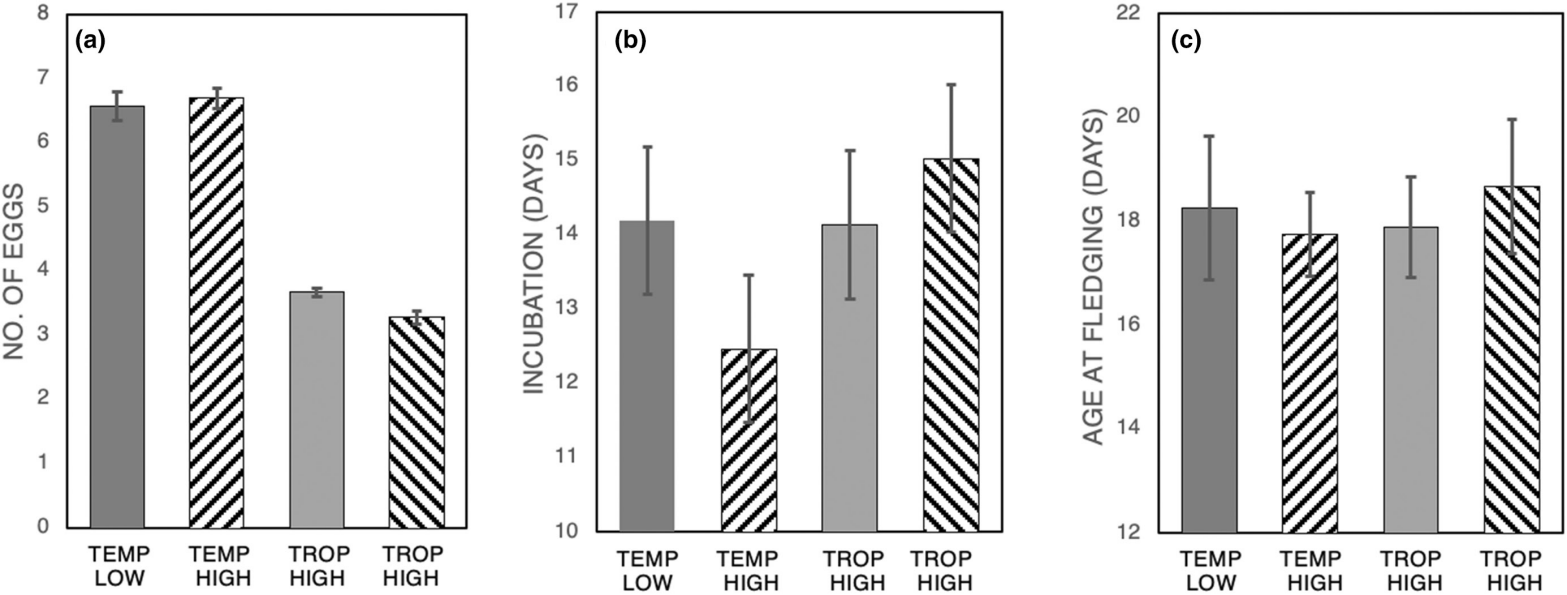
Variation (mean ± standard error) across sites in (a) clutch size, (b) duration of incubation, and (c) age at fledging.

A comparison of breeding parameters across sites within a single year showed varied effects of latitude and elevation (Figure 2, Table 2). We found a significant main effect of latitude on clutch size (Figure 2a, Table 3), with clutch size decreasing with latitude, but we found no main effect of elevation (Table 3). The latitudinal effect was qualified by a significant interaction with elevation (Table 3); thus, while clutch size decreased with latitude at both high (*p* < .001) and low elevations (*p* < .001), only tropical birds showed a significant effect of elevation on clutch size (tropical, *p* < .001, mean diff = 0.4 eggs; temperate, *p* = .511), such that high‐elevation birds had smaller clutches than low‐elevation birds.

**TABLE 2:** Mean breeding parameters across sites.

	Clutch size (eggs)	Incubation (days)	Fledging (days)	Success (% nests)	Season (days)
Temp‐low (34° N, 480 m)					
Mean	6.56	14.19	18.25	64.3	60
SE	0.15	0.24	0.28		
*n*	27	21	16		
Temp‐high (37° N, 2164 m)					
Mean	6.69	12.46	17.74	94.0	56
SE	0.14	0.21	0.23		
*n*	29	26	23		
Trop‐low (10° N, 35 m)					
Mean	3.67	14.13	17.88	45.9	>196
SE	0.10	0.17	0.22		
*n*	61	40	25		
Trop‐high (10° N, 1342 m)					
Mean	3.27	15.03	18.68	57.3	>181
SE	0.11	0.20	0.24		
*n*	49	31	22		

**TABLE 3:** Breeding phenology, incubation, and morphology two‐way ANOVA outcomes

	*df*	Latitude		Elevation		Latitude × elevation	
		*F*	*p*‐Value	*F*	*p*‐Value	*F*	*p*‐Value
Breeding parameters							
Clutch size	1, 162	633.93	<.001	1.19	.278	4.66	.032
Incubation period	1, 114	43.79	<.001	4.04	.047	41.54	<.001
Fledging period	1, 82	1.39	.241	0.36	.550	7.32	.008
Incubation							
% day incubating	1, 17	16.48	<.001	17.38	<.001	6.14	.024
No. of bouts per day	1, 17	8.09	.011	1.46	.243	0.37	.550
Bout length	1, 17	0.06	.814	0.16	.691	2.90	.107
Day nest temp	1, 17	0.74	.401	0.55	.470	8.09	.011
Day ambient temp	1, 17	24.26	<.001	106.66	<.001	18.30	<.001
Night nest temp	1, 17	0.82	.378	2.17	.159	2.34	.145
Night ambient temp	1, 17	342.07	<.001	126.13	<.001	0.38	.547
Measurements							
Male weight	1, 130	263.80	<.001	11.38	<.001	0.69	.408
Male wing cord	1, 127	9.17	.003	6.80	.010	0.66	.419
Female weight	1, 80	59.75	<.001	7.01	.010	0.06	.809
Female wing cord	1, 82	7.13	.009	9.42	.003	2.81	.098
Egg weight	1, 165	200.77	<.001	37.80	<.001	4.42	.037
Chick weight (Day 5)	1, 111	87.42	<.001	12.19	<.001	0.24	.623
Chick weight (Day 10)	1, 111	203.87	<.001	8.25	.005	4.66	.033

We found main effects of both latitude and elevation on the duration of incubation (Figure 2b, Tables 2 and 3). However, incubation was longer than expected for house wrens at our temp‐low site, resulting in a significant interaction effect between latitude and elevation on the duration of incubation (Table 3). Thus, tropic‐high birds incubated for more days than did temp‐high birds (*p* < .001, mean diff = 2.57 days), but there was no effect of latitude when comparing low‐elevation sites (*p* = .822). In addition, also as a result of the long incubation duration at our temp‐low site, we found that incubation duration decreased with elevation in the temperate zone (*p* < .001), whereas in the tropics, incubation duration increased with elevation (*p* < .001).

We found no main effects of latitude or elevation on age at fledging (Figure 2c, Tables 2 and 3), but did find a significant latitude × elevation interaction in the age at fledging (Table 3). As was the case with incubation, an effect of latitude was evident in a comparison of high‐elevation (*p* = .006), but not low‐elevation birds (*p* = .300). Within a given latitude, there was an effect of elevation on age at fledging in tropical birds (*p* = .015), but not in temperate zone birds (*p* = .161).

### 3.3 Nest predation and reproductive success

Nesting success varied across sites (mean ± SE across years: temp‐low, 77.35 ± 4.91; temp‐high, 88.5 ± 5.81; trop‐low, 50.40 ± 4.50; and trop‐high, 48.45 ± 8.85). All nests that failed after clutch completion appeared to fail due to predation at all sites. There was no evidence of death by starvation or injury; rather, evidence suggested that disappearance of eggs or chicks was due to predation by mammals (highly disturbed or destroyed nest) or snakes (undisturbed nest). When looking across years, we found a main effect of latitude on nesting success, with lower nest success in tropical birds than temperate birds (*F* = 16.501, *df* = 1, *p* = .002). There was no effect of elevation on success rate (*F* = 0.311, *df* = 1, *p* = .587), and there was no significant interaction between latitude and elevation (*F* = .631, *df* = 1, *p* = .442). Nest success at high‐elevation sites appeared to be more variable than it was at low‐elevation sites (Table 1).

Sites differed in their likelihood of successfully producing more than one clutch. For example, in the 1‐year comparison across sites, only one pair of house wrens in the temperate zone (at low elevation) successfully produced two clutches. In the tropics, at least 18% of high‐elevation pairs and 15.6% of low‐elevation pairs successfully produced more than one clutch during the period in which they were observed.

### 3.4 Incubation behavior

To examine daytime incubation behavior, we considered the activity of females between their first departure from nest box and their last return. The amount of time between when females first left and last entered the nest, a measure of active day length, showed main effects of latitude (*F* = 49.18, *df* = 1, *p* < .001) and elevation (*F* = 12.32, *df* = 1, *p* = .003), such that females were active longer at higher latitudes and elevations (temp‐low, 13.69 ± 0.24; temp‐high, 12.90 ± 0.51; trop‐low, 12.84 ± 0.84; trop‐high, 14.55 ± 0.29). There was no interaction between latitude and elevation (*F* = 0.09, *df* = 3, *p* = .767). Analysis of the average percent of the day that females spent in the nest box also showed main effects of both latitude and elevation (Tables 3 and 4), but these effects were qualified by a significant interaction effect (Table 3). This effect was due primarily to the increased time spent incubating by temp‐high females; temp‐high females spent a greater percent of their day incubating than did temp‐low females (*p* < .001), whereas tropical females showed no effect of elevation (*p* = .258). In addition, an effect of latitude on time spent incubating was only evident when comparing birds at the two high‐elevation sites (*p* < .001); the two low‐elevation sites did not differ in how much of the day was spent incubating (*p* = .268).

**TABLE 4:** Temperature and nest attendance during incubation.

	% day on nest	# bouts per day	Bout length (min)	Day nest temp (°C)	Day ambient temp (°C)	Night nest temp (°C)	Night ambient temp (°C)
Temp‐low (*n* = 6)							
Mean	44.35	30.70	12.27	25.79	22.63	21.40	15.81
SE	2.15	2.75	1.41	1.16	0.34	1.80	0.36
Temp‐high (*n* = 5)							
Mean	59.67	32.47	15.45	30.26	20.43	27.20	11.19
SE	2.36	3.01	1.54	1.27	0.37	1.97	0.40
Trop‐low (*n* = 5)							
Mean	40.70	20.50	15.20	28.26	25.98	26.10	22.77
SE	2.36	3.01	1.54	1.27	0.37	1.97	0.40
Trop‐high (*n* = 5)							
Mean	44.60	25.87	13.24	25.64	20.67	26.00	18.64
SE	2.36	3.01	1.54	1.27	0.37	1.97	0.40

The average number of incubation bouts per day showed a main effect of latitude with females at higher latitudes having more bouts than did females at lower latitudes (Tables 3 and 4). There was no significant effect of elevation on the number of incubation bouts (Table 3) and no significant interaction between latitude and elevation (Table 3). The average duration of an incubation bout did not vary with latitude (Tables 3 and 4) or elevation (Tables 3 and 4), and there was no interaction between the two factors (Table 3).

Incubation behavior is best understood within the context of ambient temperature at each site (Table 4). At night, nest box temperatures showed no main effects of latitude (Table 3) or elevation (Table 3) and no interaction effect (Table 3). However, a measure of the impact of female presence in the nest box, measured as the difference between nocturnal box and ambient temperature showed main effects of latitude (*F* = 9.11, *df* = 1, *p* = .008) and elevation (*F* = 15.95, *df* = 1, *p* < .001) and no interaction effect (*F* = 311, *df* = 1, *p* = .096). These effects were due primarily to the large impact of females on the nest at our temp‐high site (latitude: temp‐high vs. temp‐low *p* = .004, temp‐low vs. trop‐low *p* = .377; elevation: temp‐high vs. temp‐low *p* < .001, trop‐high vs. trop‐low *p* = .141).

During the daytime, ambient temperature was higher at lower latitude and lower elevation sites (Table 4). Daytime nest temperature did not show a significant main effect of latitude or elevation (Table 3), but did show a significant interaction between the two (Table 3). This was likely due to the high nest temperatures at our high‐elevation site in the temperate zone, since nest temperatures differed with elevation in the temperate zone (*p* = .019), but not in the tropics (*p* = .163), and high‐elevation nest temperatures differed between latitudes (*p* = .01), whereas low nests did not (*p* = .170). The difference between daytime average box and ambient temperature showed a main effect of latitude (*F* = 6.47, *df* = 1, *p* = .021), with a larger difference between ambient and box temperatures in the temperate zone, as well as a main effect of elevation (*F* = 17.24, *df* = 1, *p* < .001), with larger differences at higher elevations. There was no interaction effect (*F* = 3.11, *df* = 1, *p* = .096). Again, this was the result of the large impact of female presence in the nest box at our temp‐high site (latitude: temp‐high vs temp‐low *p* = .008, temp‐low vs. trop‐low *p* = .580; elevation: temp‐high vs. temp‐low *p* < .001, trop‐high vs. trop‐low *p* = .117).

### 3.5 Size and development

We began our investigation of chick development by considering the relative size of adults at our different sites (Table 5). Significant main effects indicated that adult male and female tropical house wrens weighed more than those in the temperate zone (Table 3), and house wrens of both sexes weighed more at higher than lower elevations (Table 3). There was no interaction between elevation and latitude for the weight of birds of either sex (Table 3). We also analyzed wing cord and found both main effects of latitude and elevation this variable in males (Table 3) and females (Table 3). There was no interaction effect of latitude and elevation for wrens of either sex with respect to wing cord (Table 3). Thus, house wrens of both sexes were heavier and had shorter wings at higher elevations and lower latitudes.

**TABLE 5:** Adult, egg, and chick morphological measurements.

	Male		Female			Chicks	
	Weight (gm)	Wing cord (mm)	Weight (gm)	Wing cord (mm)	Egg (gm)	Day 5 (gm)	Day 10 (gm)
Temp‐low							
Mean	9.74	51.11	10.32	49.41	1.34	4.94	9.05
SE	0.19	0.32	0.32	0.41	0.02	0.19	0.21
*n*	20	18	11	11	39	19	19
Temp‐high							
Mean	10.13	51.56	11.05	49.87	1.48	5.58	9.94
SE	0.16	0.26	0.27	0.36	0.02	0.14	0.15
*n*	28	28	15	15	36	35	35
Trop‐low							
Mean	12.11	50.14	12.33	47.99	1.63	6.52	11.94
SE	0.12	0.20	0.18	0.23	0.02	0.15	0.16
*n*	46	46	35	35	50	31	31
Trop‐high							
Mean	12.76	51.00	12.93	49.54	1.70	7.00	12.06
SE	0.13	0.22	0.22	0.28	0.02	0.15	0.17
*n*	40	39	23	25	44	30	30

House wren eggs showed a main effect of latitude (Tables 3 and 5) and elevation (Tables 3 and 5), such that eggs weighed more at lower latitudes and higher elevations. The effect of latitude on egg weight was greater than the effect of elevation, creating a significant interaction effect (Table 3).

**FIGURE 3:**
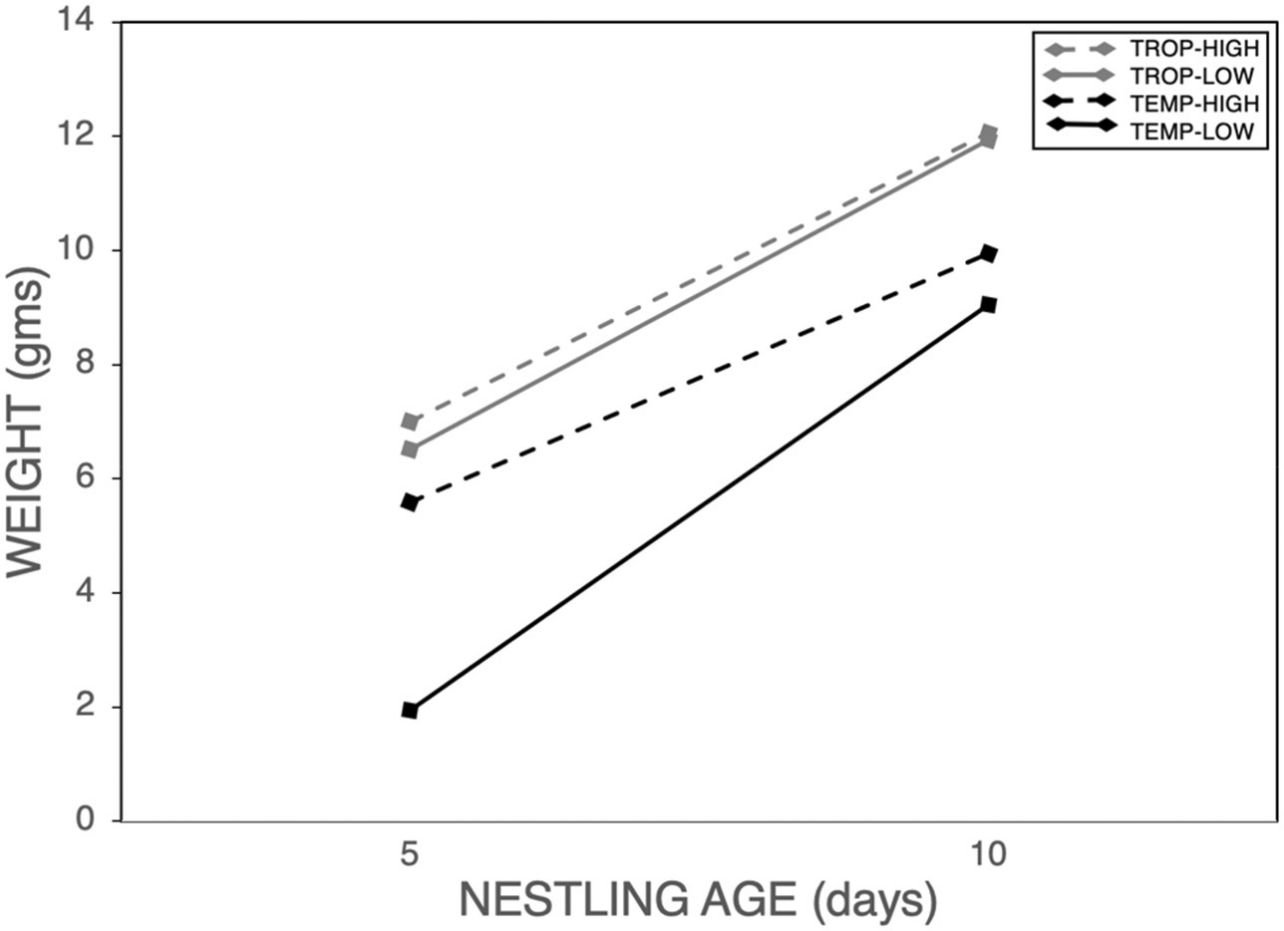
Nestling weight on Days 5 and 10 posthatch.

Nestling weights on Day 5 and Day 10 posthatching showed significant variation between sites. There was a significant main effect of latitude on the weights of 5‐day‐old chicks (Tables 3 and 5; Figure 3), such that 5‐day‐old chicks weighed more in the tropics than in the temperate zone. There was also a main effect of elevation (Table 3), with chicks weighing more at higher elevations. There was no latitude × elevation effect on Day 5 weights (Table 3). Nestling weights on Day 10 also showed main effects of latitude (Tables 3 and 5, Figure 3) and elevation (Table 3), but these effects were qualified by an interaction effect (Table 3), due to tropical birds not showing an effect of elevation (*p* = .594) whereas all other comparisons were significant (*p* < .001). Growth rate, measured by percent increase in weight between 5‐ and 10‐day‐old nestlings, did not show an effect of latitude (Table 3, Figure 3), but did show a main effect of elevation (Table 3), such that nestlings grew more slowly at higher elevations. There was no latitude × elevation effect on growth rate (Table 3).

### 3.6 Parental feeding of chicks

Feeding trips to the nest made by males and females were compared between early (Week 1) and late (Week 2) in the nestling period (Table 6). We used two measures to investigate parental feeding behavior—total number of trips to the nest and number of trips per chick. Neither showed a marked effect of latitude or elevation on parental feeding. As would be expected for the total number of feeding visits to the nest, we found a main effect of nestling age, such that parents at all sites made more trips to the nest later in the nestling period (Tables 6 and 7); nestling age did not interact with latitude (Table 7), elevation (Table 7), or show a three‐way interaction effect (Table 7). There was also a main effect of latitude on total visits to the nest, with temperate zone birds making more visits than tropical birds (Table 7) and no significant main effect of elevation (Table 7). However, these results were qualified by an interaction (Table 7), as a comparison of high‐elevation sites showed an effect of latitude (*p* < .001), whereas low elevations did not (*p* = .065). Both temperate (*p* = .008) and tropical wrens (*p* = .045) show an increase in total number of feedings with an increase in elevation.

**TABLE 6:** Mean parental nestling feeding behavior.

	Total feeds		Feeds per chick		Total male		% male	
	Early	Late	Early	Late	Early	Late	Early	Late
Temp‐low^a^ (34° N, 480 m)								
Mean	11.59	18.59	2.19	5.50	5.09	6.82	35.98	33.60
*SE*	1.46	2.24	0.35	0.54	1.22	1.65	7.51	8.00
Temp‐high^b^ (37° N, 2164 m)								
Mean	16.48	23.50	3.06	4.43	9.88	10.36	54.97	48.82
*SE*	1.06	1.62	0.25	0.39	0.89	1.20	5.44	5.79
Trop‐low^c^ (10° N, 35 m)								
Mean	9.91	13.92	3.03	4.34	5.17	7.02	49.72	56.51
*SE*	0.84	1.30	0.20	0.31	0.71	0.95	4.34	4.62
Trop‐high^d^ (10° N, 1342 m)								
Mean	6.91	11.88	2.61	4.61	3.02	5.34	42.61	48.43
*SE*	0.90	1.38	0.21	0.33	0.75	1.02	4.63	4.93

*Note*: Sample sizes were equivalent at both early and late time points as only cases with complete data could be included in the repeated measures ANOVA.

^a^ *n* = 11.

^b^ *n* = 21.

^c^ *n* = 33.

^d^ *n* = 29.

**TABLE 7:** Parental feeding of nestling two‐way ANOVA outcomes.

	Total feeds		Feeds per chick		Total male		% male	
	*F*	*p*	*F*	*p*	*F*	*p*	*F*	*p*
Time	41.47	<.001	73.89	<.001	5.42	.022	0.08	.773
Latitude	39.26	<.001	0.35	.557	11.82	<.001	1.69	.197
Elevation	1.17	.282	0.13	.722	1.78	.185	1.07	.304
Time × Latitude	1.99	.161	2.18	.143	0.52	.473	2.24	.138
Time × Elevation	0.07	.786	1.78	.186	0.08	.779	0.11	.738
Latitude × Elevation	11.40	.001	0.004	.979	12.96	<.001	7.20	.009
Time × Latitude × Elevation	0.07	.796	8.09	.006	0.40	.529	0.04	.884

When the number of feeding trips was adjusted for the clutch size (“feeds per chick”), we again found a main effect of nestling age on parental feeding behavior (Tables 6 and 7), but no main effects of latitude (Table 7) or elevation (Table 7). There was no latitude × elevation effect (Table 7). but there was an interaction of nestling age × latitude × elevation (Table 7). This effect was due to the low level of feeding by temp‐low birds in the early nestling period (Tables 6 and 7), which resulted in temp‐low birds feeding less than both trop‐low birds (*p* = .038) and temp‐high birds (*p* = .046). All other comparisons between nestling stage, latitude, and elevation were not significant at *p* > .05.

We also took a more focused look male behavior since this has been specifically predicted to increase with nestling age and elevation (Badyaev, 1997; Johnson et al., 2007). Here, we again used two measures, the percentage of the total number of visits that were made by males (when identification was possible), and the total number of visits made by males. The percentage of feeding visits made by males ranged from 33.6 to 65% (Table 6) and showed no main effect of nestling age (Table 7), latitude (Table 7), or elevation (Table 7). There was, however, a significant interaction between latitude and elevation (Table 7). Due to the low participation in feeding by the temp‐low males, the percent of feeding visits made by temp‐low males was less than that for trop‐low males (*p* = .012), as well as less than that for temp‐high males (*p* = .027). A nestling age × latitude × elevation interaction was not significant (Table 7).

The total number of visits made by males showed slightly different results than did the percentage of male visits (Table 6). We found that the number of male visits showed a main effect of nestling age (Table 7), with males increasing their number of visits with nestling age, a main effect of latitude (Table 7), and no main effect of elevation (Table 7). These effects were qualified by a latitude × elevation effect (Table 7), again due to low feeding rates by temp‐low males; temp‐high males made more feeding visits than did trop‐high males (*p* < .001), but a comparison of low‐elevation males at different latitudes was not significant (*p* = .917), and temp‐low males fed less than temp‐high males (*p* = .004), while trop‐low males fed more than trop‐high males (*p* = .048). There was not a significant nestling age × latitude × elevation effect (Table 7).

## 4 DISCUSSION

Our study of house wrens is unique in that it examines both latitudinal and elevational variation in life history traits within a single species. Moreover, we extend house wren research into the southwest and provide an extensive addition to Young's 1994 work on elevation in neotropical populations. In so doing, our data provide unexpected insights into drivers of life history strategies.

### 4.1 Variation in life history traits

Our data indicate that house wrens reproduce in shorter breeding seasons at higher latitudes; elevational differences between sites, if any, were slight (Figure 1, Table 2). Within this context, we found clutch size to be relatively consistent between years at each site, whereas the duration of incubation and fledging varied significantly between years only in the temperate zone sites. This latitudinal difference is expected given the shorter breeding season and more variable conditions in the temperate zone. Furthermore, the plasticity in incubation and fledging seems a fitting first response to the more variable conditions in the temperate zone, especially in more highly variable high‐elevation sites (Martin, 2001). Given that clutch size did not vary between years at any site, it is no surprise that it varied between sites, showing the predicted decrease in clutch size with a decrease in latitude or an increase in elevation (Figure 2a, Table 2). Although elevational differences in clutch size were significant but small, they were consistent with what was reported by Boyle et al. (2015) in their meta‐analysis of reported elevational effects on reproductive effort.

The fact that we saw interyear variability in the duration of incubation is also reflected in between site differences, namely with respect to predicted main effects that incubation duration increased with a decrease in latitude and an increase in elevation (Figure 2b, Table 2). However, these effects were qualified by an interaction effect due to unexpectedly prolonged incubation by house wrens at our temp‐low site, Marshall Canyon. Marshall Canyon also had an impact on intersite comparisons of the duration of the nestling period; that is, the prolonged nestling period evident in house wrens in Marshall Canyon was similar to what we observed at our higher elevation sites both in the temperate and tropical zones (Figure 2c, Table 2). This interesting effect is the likely reason why we did not uncover the predicted main effects of latitude and elevation in this breeding parameter.

In light of the points made above, Marshall Canyon, our low‐elevation site in the temperate zone, becomes of particular interest. House wrens have not been studied previously in Southern California, but they are common within this region and the broader southwest. In this region, house wrens seem to occur in smaller, discontinuous populations that are associated with water‐ either around the few small lakes in the region, or in riparian areas typically found in canyons (Allen et al., 2016; Levin, pers. obsn). House wrens in the southwest US experience warmer, and much drier conditions than do those in the midwestern and eastern parts of the United States (Verner & Purcell, 1999), which might affect incubation behavior, egg development (e.g., Portugal et al., 2014), and the nestling period either directly or indirectly due to possible differences in insect abundance and quality in the Southwest. The latter possibility seems more likely as an explanation for the prolonged nestling period since food supplementation near the nest accelerated fledging by 1 day early in the breeding season, but not later in the season (Levin et al., unpublished data). The periods of prolonged parental care and higher predation rate (Table 2) in Marshall Canyon make its resident house wrens similar to those at our tropical sites. However, Marshall Canyon birds also exhibit the larger clutch sizes of temperate birds. This makes the Marshall Canyon site an exciting intermediate between temperate and tropical house wrens. Given that this population is approximately 224 km from the Mexican border, it is possible that it is *T. a. brunneicollis*, found primarily in Mexico (Klicka et al., 2023), a hybrid between *T. a. brunneicollis* and *T. aedon*, the species found in most of the US, and/or evolving from a more recent arrival from Mexico. Regardless of which possibility is correct, the disconnect between having a large clutch size and prolonged periods of parental care and high predation as seen in Marshall Canyon has the potential to yield insight into which traits adapt first and how traits combine into life history strategies. We consider this issue again below when we address parental attentiveness and its impact on the duration of chick developmental stages.

### 4.2 Parental attentiveness: Incubation and feeding visits

In the temperate zone, parents of both sexes weighed more with a decrease in latitude and an increase in elevation, a pattern reported by others for this species (summarized in Johnson, 2020; Milinkovich, 1993; Table 5). In turn, egg and nestling weights showed a similar pattern, also as expected (Milinkovich, 1993; but see Evans‐Ogden et al., 2012 for a congener). Chicks grew at the same rates at different latitudes, but did so more slowly with an increase in elevation (Figure 3). Below we discuss how female incubation behavior and parental feeding of chicks might have influenced these outcomes.

Our data suggest that females adjusted their daily incubation behavior to compensate for local conditions, as has been suggested previously (Martin et al., 2018; Ricklefs et al., 2017; Table 4). The impact of females incubating during the day, measured as the nest box temperature minus the ambient temperature, was greater at higher latitudes and elevations. However, females at our temp‐low site were similar to tropical females in how much of their day was spent on the nest. Thus, our temp‐high birds, experiencing the coldest and possibly the most variable conditions, spent more time on the nest than females at any other site, so that we only saw an elevation effect in time on the nest in temperate zone birds. This also resulted in the expected latitude effect being only apparent in a comparison between temperate and tropical high‐elevation sites. The difference in time spent on the nest was not due to a difference in the length of bouts, as might be expected (e.g., Conway & Martin, 2000), but rather to the number of bouts per day.

The net result of this pattern of incubation visits across sites, coupled with the clutch mass on which females were sitting, was that nest temperature, as measured at the nest cup surface, 0.5 cm away from the eggs, did not vary significantly between sites during the day, or at night (Table 4). Our nest temperature probe was placed to maximize detection of the females arriving and leaving the box, rather than to optimize measurement of egg temperature. In addition, while the sample size in this analysis is small, it does suggest that nest temperature might not vary between sites as much as originally thought. Even if only partially true, this calls into question why the duration of incubation varies across sites. Robinson et al. (2008) conducted a common garden experiment on house wren eggs and reported that differences in the duration of the incubation period between temperate and tropical house wrens were due not to incubation temperatures, but to innate differences in the eggs themselves. In contrast, Milinkovich (1993) exchanged house wren eggs between sites of different elevations in the temperate zone and found that the foster environment caused a shift in the time eggs took to hatch. While incubation duration might be expected to be more plastic and responsive to the environment between different elevations in the same regions than between very distant populations at different latitudes, the interplay between plasticity and adaptation warrants further attention.

We also explored patterns of parental participation in feeding across sites (Table 6). Previous research has reported that, in contrast to temperate zone birds, tropical birds should make fewer visits to the nest simply as a result of their smaller clutch size. This is also predicted by the hypothesis that birds experiencing higher predation rates, such as those in the tropics, should limit their visits to the nest (e.g., Martin et al., 2000; Skutch, 1949). We found a latitudinal decrease in comparisons between feeding rates at high‐elevation sites, but not between low‐elevation sites; in other words, the low feeding rates in the temp‐low population were similar to trop‐low rates. When total feeding rates were adjusted by brood size, house wrens did not generally vary in the number of feeds per chick, again with the exception of very low rates of feeds per chick at our temp‐low site. This suggests that the slower growth rate we observe in tropical birds at high versus low elevations (Figure 3) may be due to differences in food quality, since rate of feeds per chick does not differ. The fact that temp‐high birds grew more slowly than temp‐low birds even though temp‐high birds fed their chicks more suggests either an even more pronounced effect of food quality or that an additional unknown factor may be at play. One such factor is the high predation rate at our low‐temp site, which may drop their feeding rate relative to that seen at the temp‐high site.

Previous research also predicts that male participation in feeding should increase with nestling age and an increase in elevation (Badyaev, 1997; Johnson et al., 2007). We found no evidence that the proportion of all feeding visits performed by males changed with nestling age (Table 6). However, the proportion of visits made by males at the temp‐low site was low enough that it was significantly less than the trop‐low birds to which they were compared, even though the temp‐low clutch size was larger. The proportionately low male participation in feeding at our temp‐low site resulted in a comparatively higher level of male participation at the temp‐high site. A similar comparison of different elevations in the tropics was not significant, supporting the suggestion that males do not increase their participation with an increase in elevation.

It has been argued that the absolute number of visits made by males is a better measure of male participation, in part because the proportional measure can be affected by female, as well as male behavior, as was seen in mountain bluebirds (Johnson et al., 2007). Using this absolute measure, males did increase their number of feeding visits with nestling age (Table 6). Other comparisons yielded similar results to the proportional visits and reflected the unexpected low participation of males at our temp‐low site. Another benefit of using an absolute rather than a relative measure is that it allows for a test of the hypothesis that birds experiencing high predation pressure should make fewer visits to nest, thus feeding less. We found this to be the case for male feeding visits; the temp‐low males were similar to the trop‐low males, both in the number of male feeding visits and high nest predation levels.

The findings on incubation and feeding behavior add to our understanding of how best to view the temp‐low birds in Marshall Canyon. In addition to showing tropical patterns in the duration of incubation and nestling periods, they also show evidence of a reduced number of feeding visits at levels that would be expected in tropical birds. However, given the large clutches of the temp‐low birds, the low number of feeding visits by temp‐low wrens is more likely the result of either low food availability (but see above) or higher food quality.

### 4.3 Life history hypotheses

Our results on tropical house wrens confirm a suite of traits that characterize a strategy that includes a longer breeding season with multiple breeding opportunities per pair, bigger adults with shorter wings, smaller and fairly stable clutch size, larger eggs and nestlings, longer periods of incubation and fledging, less time per day spent in incubation, and fewer feeding visits per nest. Our ability to compare tropical wrens directly with our temperate populations was complicated by the fact that our low‐elevation temperate zone site differs from other sites in the US in which house wrens have been studied (e.g., Ohio, Illinois, New York). However, latitudinal comparisons between high‐elevation sites yielded results consistent with expectations.

House wrens at our tropical sites also provided an opportunity to compile a broad array of traits that vary with elevation, adding the Neotropics to the small list of regions in which elevation effects have been studied (Boyce et al., 2015). We found that an increase in elevation in tropical wrens is accompanied by a larger body and shorter wings, larger eggs and nestlings, smaller clutch sizes, longer incubation and nestling periods, slower nestling growth, higher nest success, and cooler temperatures. There was no impact of elevation on time spent incubating or amount of feeding when adjusted for clutch size.

In sum, the suite of traits associated with a decrease in latitude and an increase in elevation mirror each other. An exception to this is that nest success increases with an increase in elevation, but decreases with latitude. Datasets from a single species or groups of very closely related species (Klicka et al., 2023) are a powerful way to test ideas about suites of traits that group together composing a strategy, but are insufficient by themselves to exclude different hypotheses about which selective pressures might explain these patterns without direct manipulations. Data from Robinson et al. (2008) and Wikelski et al. (2003) on latitudinal effects and Milikonovich (1993) on elevational effects are enticing in this regard.

Our temperate zone birds are of particular interest because they extend house wren research into the western US, particularly at low elevations. Birds at our high‐elevation site align in many ways with those found by the other comprehensive studies of high‐elevation house wrens in Wyoming (Johnson, 2020), Colorado (Milinkovich, 1993), and Arizona (Cheng & Martin, 2012; Palacios & Martin, 2006). All high‐elevation house wren study sites are in the West; the addition of high‐elevation populations in the eastern US would add insight into variation and driving forces behind suites of traits.

The temp‐low site, Marshall Canyon, yielded provocative results. Marshall Canyon house wrens had large clutches, smaller adults with longer wings, and smaller eggs and young, like most temperate zone birds that have been studied. However, Marshall Canyon wrens also had longer incubation and nestling periods, less time on the nest, lower feeding rates, and low nest success rates, which are more typical of tropical birds. It is unclear whether these differences are due entirely to local conditions, a different evolutionary history and taxonomic status than house wrens further east, or both. Of the selective pressures proposed to drive different strategies, food limitation would seem a likely explanation of the lower rates of female incubation and parental food delivery and at least one unpublished food supplementation study (Levin et al., unpublished data) suggests that this may be the case. A high predation rate, leading to a strategy of fewer visits to the nest to incubate and feed young, is another possible explanation. Testing between selective pressures at this and other sites in this region and comparisons with other Mexican (*T. a. brunneicollis*) and tropical populations (*T. a. musculus*) is essential.

House wrens provide an almost unrivaled opportunity to unravel the contribution of different ecological and physiological adaptions to the evolution of life history strategies. Results of our study of populations at different elevations in California and Costa Rica suggest that additional observational and experimental studies at a broader diversity of sites have the potential to add powerful insights to our understanding of life history evolution.

## AUTHOR CONTRIBUTIONS

**Rachel N. Levin:** Conceptualization (lead); data curation (equal); formal analysis (lead); funding acquisition (lead); investigation (lead); methodology (lead); project administration (lead); resources (lead); software (lead); supervision (equal); validation (lead); visualization (lead); writing – original draft (lead); writing – review and editing (equal). **Stephanie M. Correa:** Data curation (equal); investigation (equal); methodology (supporting); project administration (equal); resources (supporting); software (supporting); supervision (equal); validation (supporting); visualization (supporting); writing – original draft (supporting); writing – review and editing (equal). **Kate A. Freund:** Conceptualization (supporting); data curation (equal); formal analysis (equal); funding acquisition (supporting); investigation (equal); methodology (supporting); project administration (equal); resources (supporting); software (supporting); supervision (equal); validation (supporting); visualization (supporting); writing – original draft (supporting); writing – review and editing (supporting). **Matthew J. Fuxjager:** Conceptualization (supporting); data curation (supporting); formal analysis (supporting); funding acquisition (supporting); investigation (equal); methodology (supporting); project administration (equal); resources (supporting); software (supporting); supervision (equal); validation (supporting); visualization (supporting); writing – original draft (supporting); writing – review and editing (equal).

## FUNDING INFORMATION

Student participation on this project was supported by the Pomona College Summer Research Program and the NSF Research Experiences for Undergraduates Program.

## CONFLICT OF INTEREST STATEMENT

The authors have no conflicts of interest. The findings and conclusions in this article are those of the authors and do not necessarily represent the views of the U.S. Fish and Wildlife Service.

## Data Availability

Data described in this paper are accessible via the Dryad data repository at 10.5061/dryad.547d7wmbp.
